# Health reform and Indigenous health policy in Brazil: contexts, actors and discourses

**DOI:** 10.1093/heapol/czaa098

**Published:** 2020-11-09

**Authors:** Ana Lucia de M Pontes, Ricardo Ventura Santos

**Affiliations:** c1 Departamento de Endemias Samuel Pessoa, Escola Nacional de Saúde Pública, Fundação Oswaldo Cruz/FIOCRUZ, Rio de Janeiro, Brazil; c2 Departamento de Antropologia, Museu Nacional, Universidade Federal do Rio de Janeiro, Rio de Janeiro, Brazil

**Keywords:** Indigenous health, health reform, history, qualitative research

## Abstract

Given the challenges related to reducing socio-economic and health inequalities, building specific health system approaches for Indigenous peoples is critical. In Brazil, following constitutional reforms that led to the universalization of health care in the late 1980s, a specific health subsystem was created for Indigenous peoples in 1999. In this paper, we use a historical perspective to contextualize the creation of the Indigenous Health Subsystem in Brazil. This study is based on data from interviews with Indigenous and non-Indigenous subjects and document-based analysis. In the 1980s, during the post-dictatorship period in Brazil, the emergence of Indigenous movements in the country and the support for pro-Indigenous organizations helped establish a political agenda that emphasized a broad range of issues, including the right to a specific health policy. Indigenous leaders established alliances with participants of the Brazilian health reform movement, which resulted in broad debates about the specificities of Indigenous peoples, and the need for a specific health subsystem. We highlight three main points in our analysis: (1) the centrality of a holistic health perspective; (2) the emphasis on social participation; (3) the need for the reorganization of health care. These points proved to be convergent with the development of the Brazilian health reform and were expressed in documents of the Indigenist Missionary Council (CIMI) and the Union of Indigenous Nations (UNI). They were also consolidated in the final report of the First National Conference on the Protection of Indigenous Health in 1986, becoming the cornerstone of the national Indigenous health policy declared in 1999. Our analysis reveals that Indigenous people and pro-Indigenous groups were key players in the development of the Indigenous Health Subsystem in Brazil.

**KEY MESSAGES**

- Over the past decade, several studies have analysed the political conditions that allowed for the emergence and consolidation of a national health policy for Indigenous peoples in Brazil.
- However, there is limited content describing the context, discourses and actors involved in this process.
- The Brazilian case shows that health policies aimed at Indigenous peoples have emerged from a complex combination of events, meanings, social relations and subjects agencies.
- Alliances between the Indigenous movement and the health reform movement were central for this process.

## Introduction 

Historically, Indigenous peoples have experienced marginalization, exclusion and discrimination (Horton, 2006; Gracey and King, 2009; Anderson *et al.*, 2016). In Latin America, existing evidence also demonstrates pronounced health inequities between Indigenous and non-Indigenous people across the region (Montenegro and Stephens, 2006; Anderson *et al.*, 2016).

In 2007, the United Nations (UN) decreed the first Declaration on the Rights of Indigenous Peoples, which acknowledges the urgent need to implement initiatives that promote the respect and protection of Indigenous peoples’ rights worldwide (UN, 2007). It also emphasizes Indigenous peoples’ right to health services, the importance of their active participation in the formulation and implementation of health programmes, and their right to maintain traditional medicines and health practices. The UN Declaration also asserts that national governments must implement necessary measures to promote the health of Indigenous peoples.

We argue in this paper that an exploration of the Brazilian context can contribute to the international debate on reforms related to Indigenous peoples’ health care. Constitutional reforms that took place in Brazil during the late 1980s established mandatory universal provision of health care (Paim *et al.*, 2011). The new constitution, declared in 1988, also determined that the Brazilian State must recognize the sociocultural and territorial rights of Indigenous peoples (Ramos, 1998; Cunha, 2018). These milestones also led to the formulation of a specific Indigenous health policy. In 1999, the Arouca Law created an Indigenous Health Subsystem within the Brazilian national health system (FUNASA, 2002; Langdon, 2010; Coelho and Shankland, 2011; Garnelo, 2014). In Latin America, Brazil was one of the first countries to propose a specific national health policy for Indigenous peoples (Langdon and Cardoso, 2015).

It is currently estimated that Brazil’s Indigenous population is of ∼900 000 people. Representing 0.4% of the national population, Indigenous peoples of Brazil have remarkable socio-diversity, with nearly 300 different ethnic groups speaking ∼270 different Indigenous languages (Santos *et al.*, 2019). Research has consistently revealed unfavourable sociodemographic and epidemiological indicators for Indigenous peoples in Brazil (Anderson *et al.*, 2016; Campos *et al.*, 2017; Santos *et al.*, 2020).

The Brazilian Indigenous Health Subsystem serves the Indigenous population living in Indigenous territories through a structure of 34 local health-care units, called Indigenous Special Health Districts (DSEI). Each district is responsible for delivering primary care with multidisciplinary health teams. These teams include Indigenous community health workers, doctors, nurses, dentists and oral health technicians. This health-care model is based on the notion of ‘differentiated health care’, which advocates that health initiatives should consider the linguistic, sociocultural and geographical specificities of Indigenous territories. Furthermore, health actions should be undertaken in dialogue with Indigenous peoples to ensure Indigenous knowledge, practices and specialists are embedded in health programmes and policies. Each district has a participatory structure called the Indigenous Health District Council (CONDISI). This subsystem seeks to address both the marginalization and health inequalities that affect Indigenous peoples, as well as the challenges of developing a health-care model that considers diversity and sociocultural specificities (Coelho and Shankland, 2011; Cardoso *et al.*, 2012; Mendes *et al.*, 2018).

The subsystem was created in 1999, by the Arouca Law, but the specific aspects of the health-care system were only established in 2002 through the ‘National Policy for the Health Care of Indigenous Peoples’ (PNASPI) (FUNASA, 2002). This document presents a narrative on the construction of the Indigenous Health Subsystem, and states:

*The elaboration of the National Policy for the Health Care of Indigenous Peoples included the participation of representatives from the bureaus responsible for health policies and the government's Indigenous policy and action, as well as civil society organizations with renowned trajectories in the field of care and human resources training for the health of Indigenous peoples. (…) the elaboration of this proposal included the participation of representatives from Indigenous organizations with experience in executing projects in the field of health care alongside their people* (FUNASA, 2002, p. 6).

In the text above, the ‘participation’ of members of non-governmental organizations and representatives of Indigenous peoples is explicitly mentioned, even though they are not depicted as protagonists of the policy-making process. There is also a brief presentation of the trajectory of governmental actions regarding the health of Indigenous peoples, and the landmarks of the new Indigenous health policy: The First National Conference on Indigenous Health Protection (1st CNPSI) (1986) and the Second National Health Conference for Indigenous Peoples (2nd NHCIP) (1993). Finally, the complete document emphasizes the decrees, resolutions and legal frameworks for the implementation of the Indigenous health subsystem (FUNASA, 2002).

Over the past decade, several studies have analysed the political conditions that allowed for the emergence and consolidation of a national health policy for Indigenous peoples in Brazil (Athias and Machado, 2001; Langdon, 2010; Coelho and Shankland, 2011; Teixeira and Garnelo, 2014; Langdon and Cardoso, 2015). It is worth noting that very few studies carried out so far have emphasized the roles played by Indigenous movements and non-Indigenous advocates in the creation of the contents and directions of the policy (Verani, 1999; Langdon, 2010). Even so, the emphasis of these studies has been on Indigenous participation during the time period that followed the formulation of the Indigenous Health Subsystem in the late 1990s (Langdon and Diehl, 2007; Cardoso *et al.*, 2012; Diehl *et al.*, 2012; Garnelo, 2012, 2014).

The First National Conference on Indigenous Health Protection (1st CNPSI), which was held in 1986, is recognized as a major event in the creation process of the Brazilian Indigenous Health Subsystem (Langdon, 2010; Cardoso *et al.*, 2012; Garnelo, 2012, 2014). In the literature, there are many references to the institutional dispute between governmental organizations (the National Indian Foundation—FUNAI and the National Health Foundation—FUNASA), the several legal decrees in the process and the consolidation of the proposal at the Second National Health Conference for Indigenous Peoples (2nd NHCIP) in 1993 (Verani, 1999; Garnelo, 2006, 2014; Langdon, 2010; Cardoso *et al.*, 2012). However, there is limited content describing the context, discourses and actors involved in this process. In addition, there has been little discussion on the conditions that articulated the Indigenous rights struggle with the national sanitary reform of the late 1980s.

In this paper, we aim to situate and contextualize the actors and socio-political processes present in the formulation of Brazil’s Indigenous health policy, as we argue that they have only been present ‘between the lines’ of the usual narratives. Under this perspective, ‘participation’ is not only involvement in institutionalized spaces, but a multiple and complex network of actors, discourses and events related to public policy-making processes (Bernstein, 2017). By reconstructing this historical process through the analysis of documents and interviews, we intend to unveil the long-term development and multiplicity of social actors involved in the creation of the Indigenous Health Subsystem in Brazil. Based on the interview data and documents analysed, we argue that Indigenous leaders and their supporters played important roles in the establishment of the Indigenous Health Subsystem in Brazil. Our analysis points out that the formulation of the policy was grounded in the context of struggles for the rights of Indigenous peoples in the 1980s, along with the emergence of the Indigenous movement. Moreover, there were important intersections between health reform and Indigenous movements.

## Methods

The data collection began in March 2018 and has involved interviews and gathering documentary material at a national level. We conducted open-ended interviews with Indigenous leaders and non-Indigenous actors, our intent being to characterize and contextualize the socio-political backgrounds and debates from the 1970s to the 1990s, which then resulted in the establishment of the Indigenous Health Subsystem in Brazil.

The interviews were informed by an oral history perspective, each lasting between 90 and 150 min. The participants recalled their experience with Indigenous health issues based on a pre-defined list of points. Initially, the first author of this paper contacted the subjects, presented the goals of the project and, once they agreed to participate, defined a place and time for the interview. Everyone who was invited agreed to be interviewed. Interviews were audio-recorded and guided by the first author, who participated in all stages of the project, and who has researched Indigenous health for the past 12 years, therefore being very familiar with the narratives regarding this policy and some of the interviewees.

All interviewees received material about the project and research team, and also signed the Informed Consent Form. The transcribed interviews were initially read by two independent researchers (the two authors), with the objective of identifying a set of broad and encompassing themes for further analyses. We used Atlas.ti^®^ to facilitate the identification of themes. In addition, the interviewees’ quotes have been partially edited for clarity.

For the purposes of this paper, we focused on a set of social actors and documents related to the historical landmarks of the Indigenous Health Subsystem prior to its creation in 1999, which were the First National Conference on Indigenous Health Protection (1st CNPSI) in 1986, and the Second National Health Conference for Indigenous Peoples (2nd NHCIP) in 1993. We reviewed the official reports of these events and interviewed members of their drafting committees. Other relevant documents and social actors were mentioned in the special supplement of the publication *Saúde em Debate* from 1988, presenting different authors’ text contributions to the discussions of the 1st CNPSI (CIMI, 1988; UNI, 1988). Finally, we selected a subset of six interviews which refer directly to the time period and issues that are of interest to this paper.

A brief introduction of the six interviewees follows:

Ailton Krenak is a prominent Indigenous leader of the Krenak people from the state of Minas Gerais, member of the Union of Indigenous Nations (UNI) and member of the committee that prepared the final report of the 1st CNPSI. Douglas Rodrigues is a physician from the Federal University of São Paulo who has worked since 1981 in several health initiatives at the Xingu Indigenous Park, located in the north of the state of Mato Grosso, and is mentioned in the final report of the 2nd NHCIP. Marcos Pellegrini is a physician from the Federal University of São Paulo who worked in the provision of health services among the Yanomami in the state of Roraima, and was involved in the report committee of the 2nd NHCIP. Mirthes Versiani is a nurse who worked for the Indigenist Missionary Council (CIMI), co-ordinated health actions for this entity in several regions and attended the 1st CNPSI and 2nd NHCIP. Zezinho Kaxarari is an Indigenous leader of the Kaxarari people and was a member of several Indigenous organizations in the state of Acre, particularly the Union of Indigenous Nations in Acre (UNI-Acre) since the 1980s. Zezinho was a representative of the Indigenous population in national committees such as the Intersectoral Commission on Indigenous Health (CISI) and the National Health Council, and participated directly in the implementation of two Special Indigenous Health Districts in Acre. Alba Figueroa is an anthropologist who worked directly with the Indigenous movement in the 1980s, formulated and developed health projects for Indigenous peoples in the Alto Rio Negro region and worked at the National Health Foundation/Ministry of Health during the implementation of the DSEIs. The researchers who conducted the interviews met the interviewees at workplaces, homes or reserved spaces in the cities of São Paulo, Rio de Janeiro, Brasília, Boa Vista and Serra do Cipó, all located in Brazil.

Furthermore, in this paper, we aim to reconstruct the historical narrative using a content analysis perspective, and selected themes for discussion, which emerged in the documents and interviews. The content of the interviews was selected and interpreted based on the published anthropological and public health literature about the trajectory of the Indigenous movement and the emergence of the Indigenous Health Subsystem in Brazil over recent decades.

## Results and discussion

### Resistance to the dictatorship and the emergence of new social actors

Recognizing that health policies emerge in specific socio-historical contexts (Shore and Wright, 2011; Bernstein, 2017), we found that the formulation of the current Indigenous health policy was closely linked to the democratization of the country after two decades of military dictatorship (1964–85). This process was consolidated with the proclamation of the 1988 Constitution but had already begun in the second half of the 1970s. During this period, despite the censorship and repressive measures imposed by the authoritarian state, the defence of Indigenous peoples became an agenda for the mobilization of civil society, uniting Indigenous peoples, anthropologists, doctors and other actors (Cunha, 2018).

For example, an Indigenous leader at the forefront of this political struggle reported:

*In the 1960–70s, Brazilian State policies were already spreading the idea that the Indians, if they still existed, had already been contacted and were close to becoming integrated into national society. In the 1970s, the ministers of the military dictatorship inaugurated a project that they called ‘emancipation.’ Emancipation would be a kind of final act in which the right of the Indians to exist as a distinct social group would be resolved with their full integration into national life. It is also worth mentioning that […] on the side of civil society, resisting the dictatorship’s actions, there was a social movement that was shouting, that was throwing into question the state’s assertion that Indians could now be emancipated* (Ailton Krenak).

As is evident in this interview, the catalytic event for the debates on Indigenous policy was the proposal for the so-called ‘Emancipation Decree’ in 1978, by Interior Minister Mauricio Rangel Reis (Ramos, 1998; Cunha, 2018). According to anthropologist Alcida Ramos, ‘Emancipation, in this special and deceptive interpretation, meant the termination of the Indian’s special status (…). It became clear that to emancipate the Indians meant, and still means, to emancipate their inalienable lands and open them for sale’ (Ramos, 1998, p. 80).

The oppositional response to the ‘Emancipation Decree’ mentioned by Krenak led to the consolidation of an Indigenous movement and the creation of several non-governmental pro-Indigenous organizations supporting their struggle, such as the Commission for the Creation of the Yanomami Park (CCPY) in 1978, the National Association for Indigenist Action (ANAI) in 1978 and the Pro-Indian Commission (CPI) in 1979 (Cunha, 2018). These entities started working closely with Indigenous leaders to make their demands more visible and provide technical assistance for their claims, especially in the process of demarcating Indigenous territories, but also implementing health-care actions, since FUNAI was not able to provide these to all regions.

According to a health professional who worked with the Yanomami in the Amazon region:

*There was no organized health care service. Since 1982, CCPY had been concerned with providing a health service, especially vaccinating everyone, because they had a very traumatic experience [a measles epidemic] with the opening of the Perimetral Norte Highway in the 1970s. So, of course, I organized a census and vaccinated as many people as possible* (Marcos Pellegrini).

As underscored by this interviewee, an important strategy for increasing the visibility of Indigenous demands was the production of information on the demographics and health status of Indigenous peoples (Santos *et al.*, 2019). While the Brazilian government’s Indigenous policy was based around the paradigm of their eventual disappearance, various institutions sought to ‘put the Indians on the map’ (Santos *et al.*, 2019). Particularly anthropologists, most working in academia, reported in the interviews that during their research they had found evidence of epidemics and Indigenous genocide resulting from development projects in the Amazon. The anthropologists denounced this development and called for state action to protect Indigenous territories and provide health care for their populations (Davis, 1977; Ramos, 1993, 1998).

These alarming health scenarios in Indigenous communities led to an increasing interaction between Indigenous leaders, pro-Indigenous organizations and health professionals, particularly doctors. These professionals implemented health actions in Indigenous territories in various regions and constituted a support group for Indigenous health debates.

It was also in the 1970s that debate in Latin America led to the ‘Declaration of Barbados’, and the emphasis that governments needed to recognize the right of Indigenous peoples to live according to their own traditions and cultures, guarantee their territorial rights and respect their self-determination (Bartolomé, 2017). This declaration also questioned the role of missionaries, which in Brazil resulted in the creation of the Indigenist Missionary Council (CIMI) in 1972. Inspired by the liberation theology perspective that greatly influenced the Catholic Church in Latin America in the 1960s (Ramos, 1998), CIMI aimed to promote a new type of missionary action within the Brazilian Catholic Church, seeking to break with the traditional model of evangelization in order to support Indigenous communities in the defence of their territories. Several interviewees refer to connections with CIMI, which operated throughout the national territory and developed various health actions. Between 1978 and 1988, CIMI promoted several events to discuss Indigenous health, creating the conditions for increasing interactions between Indigenous and pro-Indigenous actors.

CIMI was also cited in several interviews as an entity that promoted Indigenous participation and political organization, given that since 1974 it had promoted the so-called ‘Indigenous Assemblies’ (Ramos, 1998). While these were initially stimulated by the CIMI, they were soon appropriated by the Indigenous communities:

*Our leaders held a general meeting to discuss all the topics, and NGOs like CIMI and the Pro-Indian Commission, along with FUNAI, sometimes helped. And we thought we could do it; we could do it ourselves, fight for the right to represent our own people* (Zezinho Kaxarari).

These assemblies allowed Indigenous leaders from different regions to meet and discuss a common agenda. This process led to the creation, in 1980, of the first national entity of Indigenous representation in Brazil, the Union of Indigenous Nations (UNI). UNI’s stated purpose was to establish a new relationship between Indigenous peoples and the government, especially with the aim of ensuring the protection of Indigenous lands (Ossami, 1993).

When the debates about the new constitution initiated in the second half of the 1980s, the Indigenous movement and pro-Indigenous organizations came together to propose a popular amendment for the rights of Indigenous peoples. The constitutional text was reinforced by the significant presence of Indigenous representatives in the National Congress. Furthermore, it consolidated a new relationship between Indigenous peoples and the Brazilian State (Bicalho, 2010), as pointed out by Ailton Krenak:

*The debate in the Constituent Assembly of 1987 was the moment when many Indigenous people from different groups were able to vocalize their view of the Brazilian State. Indigenous peoples began to demand their right for respect and recognition of their historical territories. This created a deep connection between identity and territory* (Ailton Krenak).

Article 231 of the 1988 Brazilian Constitution recognizes Indigenous peoples’ rights to their social organization, customs, languages, beliefs, traditions and lands they traditionally occupy (Cunha, 2018). This constitutional statement made possible the discussion of a specific public health policy for these populations. If Indigenous peoples were facing extinction in the 1970s, during the 1980s they managed to construct ideas and demands on citizenship and the right to a specific health policy:

*Up to that moment [until the 1990s], we believed that health was our problem […] but the dispute with the state [during the constituent process] presented policies and the field of health care in other terms. It is when this [Indigenous] population begins to position itself as citizens, as subjects of rights, that the state has to dialogue with their demands* (Ailton Krenak).

### Intersections between Indigenous struggles and the Sanitary Reform Movement

The complex and multi-faceted intersections between Indigenous rights struggles and the health reform movement were also addressed in several interviews, allowing for the understanding of the convergence of contexts, discourses and actors.

Law 9836 of 1999, which instituted the Indigenous Health Subsystem in Brazil, is also called the Arouca Law. It received this name in recognition of the work of federal congressman Sergio Arouca, who first introduced the bill in 1994. Sergio Arouca (1941–2003) was a physician and public health researcher at the National School of Public Health of the Oswaldo Cruz Foundation. He was a key figure in the debates that resulted in Brazilian health reform (Escorel, 2015). Acknowledging this fact, our investigation also aimed to undertake an in-depth exploration of the relationship between the Indigenous movement and health reform that may initially seem unrelated.

The so-called health reform movement pushed for the reformulation of the national health system and for the universal right to health. This process began in the 1970s through the actions of civil society organizations in resistance to the dictatorship, including social movements, health professionals and academia (Cohn, 1989; Paim, 2008).

In the interviews with health professionals, particularly physicians who worked for Indigenous peoples, informants stated that these groups had also been actively involved in the debates on Brazilian health reform. Throughout the 1980s, the health reform movement organized various debates and initiatives that addressed the reorganization of health services. These proved fundamental for the dissemination of their ideas, and for the training and interaction with a broad range of health professionals, community leaders and academics committed to the health reform initiative (Escorel, 1999). In the perspective of a physician who worked in the Xingu Park:

*So, this whole thing that we started in the Xingu Indigenous Park had everything to do with the ideas of the health reform movement itself. I mean, we actively participated in the health reform movement. All that discussion about local health systems, health districts, the centrality of primary care, community participation, all these ideas that were being discussed in Latin America and Brazil during the health reform movement—I mean, we learned it all* (Douglas Rodrigues).

At the same time, pro-Indigenous organizations sought partnerships with public health institutions like the Paulista School of Medicine (at present the Federal University of São Paulo—UNIFESP) and the Oswaldo Cruz Foundation (FIOCRUZ), in order to receive advice on the implementation of health actions and the analysis of the health situation of Indigenous peoples. A set of actors involved with health reform were called on to support the Indigenous agenda, and therefore played a strategic role in translating Indigenous interests and discourses into the health reform debate.

*The district’s proposal happened exactly in a meeting [The CIMI’s Meeting on Indigenous Health], because I would ask for a lot of assistance, I would ask the Paulista School of Medicine, there was also FIOCRUZ, Doctor Marcos from the Amazon, and they did everything… so I would always ask for their advice. This allowed us to improve our vision and make it more technical, something we previously did not have* (Mirthes Versiani).

Since the 1970s, Indigenous leaders and their allies had been highlighting FUNAI’s weakness in the implementation of health actions. Moreover, FUNAI itself organized three meetings between 1984 and 1985 whose reports indicated the need for a new health policy for Indigenous peoples (Pontes *et al.*, 2019). This period coincided with the organization of the largest ever health reform event: the 8th National Health Conference (8th CNS), held in March 1986. Debates on the democratization of health led to a broad civil society participation in the conference.

The 8th CNS, which had Arouca as its president, was perceived as preparation for the constitutional debates because the drafting committee for the new constitution had already been created in 1985 and would begin working in 1987 (Escorel, 2015). As a result, the final report on the 8th CNS constituted the text approved as the Health Chapter of the new 1988 Constitution. It was this conference that our interviewees identified as the main event of co-ordinated action, organized by Indigenous leaders and their allies to hold a specific meeting to discuss a new Indigenous health policy. In November 1986, the First National Conference on Indigenous Health Protection (1st CNPSI) took place with the participation of Indigenous organizations and their leaders.

*In 1986, at the 8th National [Health] Conference in Brasília, Indigenous leaders were already participating, and this was where they questioned the health situation of the Indigenous populations in Brazil. We wanted to have discussions and hold a meeting to assess the health status of the Indigenous population* (Zezinho Kaxarari).

The organization of the 1st CNSPI is perceived as a thematic event integrated into the 8th National Health Conference, and therefore part of the national health reform discussions (Arouca, 1986). It is considered the groundbreaking milestone for the construction of the Indigenous Health Subsystem and its guidelines. After this conference, discussion on Indigenous health was articulated with events and debates about the general health-care reform—an articulation that proved successful because they shared common concerns and discourses. However, there were still specificities to be guaranteed.

*When the group I was closest to was invited to participate in the ‘Eighth National Health Conference,’ I understood that the existing rough draft simply could not capture our idea of what care meant, that it could not be captured by the design of the universal health care system [SUS]. There had to be a subsystem, and that is why I demanded that it be called an ‘Indigenous’ health conference* (Ailton Krenak).

Besides the confluence of context and actors, we highlight three discursive dimensions of the health reform that converged with Indigenous demands and specificities: (1) the centrality of a holistic health perspective; (2) an emphasis on social participation; and (3) the need to reorganize health care. For the purposes of this analysis, we focused on the documents containing the proposals for the 1st CNPSI, formulated by UNI and CIMI, and published in *Saúde em Debate* in 1988, as well as the conference’s final report from 1986.

The health reform movement proposed a reconfiguration of the health concept, seeking connections with the broader social-historical dimensions of society, discussing medical practices and rearticulating medical assistance and public health dimensions (Cohn, 1989; Arouca, 2003; Paim, 2008). These debates were formulated in dialogue with international debates, such as those on Primary Health Care (WHO, 1978), Social Medicine in Latin America (Laurell, 1982; Nunes, 2006) and Preventive Medicine (Arouca, 2003). The centrality of the so-called ‘holistic’ concept of health underscores how it is socially determined (Paim, 2008; Escorel, 2015), a dimension important to the views of Indigenous peoples (King *et al.*, 2009). The UNI’s contributions to the 1st CNPSI emphasize the socio-diversity of Indigenous peoples in Brazil, and their struggle to have the government acknowledge their self-determination in social, economic and political dimensions (UNI, 1988). Therefore, guaranteeing the demarcation and protection of their lands emerges as a fundamental condition for their right to health. These dimensions are also defended in the CIMI’s document, which gathered the historical context behind invasions and disrespect of Indigenous lands, as well as the legal background that supports Indigenous rights (CIMI, 1988).

The report of the 1st CNPS states that ‘the health of Indigenous nations is determined by a historical time and space, by the particularity of their contact with national society, and by the form of occupation of the Indigenous territory and its surroundings’. Consequently, it implies the ‘autonomy, territorial ownership and exclusive use by Indigenous nations’ of their lands (CNPSI, 1986, p. 1). For the Indigenous movement, there was clearly an intrinsic relationship between health and land rights.

The centrality of ‘holistic’ health was an important discursive intersection for the negotiation of Indigenous health specificities in the health reform, including ‘respecting and recognizing the specific health care practices of Indigenous nations’ (CNPSI, 1986, p. 2). This also supported the creation of an Indigenous health subsystem, and the guidelines for differentiated health care. As pointed out by Zezinho Kaxarari:

*Up to 1988, we already knew that the public health system would never meet the real needs of the Indigenous populations; it would need to have some mechanism, something of its own, a system in itself that could serve our population. It had to consider our specific rights. Assist us as we are* (Zezinho Kaxarari).

Paim (2008) points out that the Brazilian health reform was a political movement, based on the conception that health reform was subordinated to the broader goals of society, such as democracy and human rights. The struggle for democracy was an important part of the health reform movement, and Indigenous leaders and allies also understood that the new Indigenous health policy implied constitutional rights, which also meant to overcome the government’s model of guardianship over them. The 1st CNPSI report stated that ‘full citizenship, ensuring all constitutional rights, is recognized as a determinant of health’ (CNPSI, 1986, p. 1).

Debates on the democratization of health also developed into the demand for broad social participation in the formulation and implementation of health policies. UNI and CIMI’s documents mention the need for ‘Indigenous participation’ (CIMI, 1988; UNI, 1988). Therefore, the report from the 1st CNPSI proposes to: ‘Guarantee the participation of Indigenous nations, via their representatives, in the formulation of policies, planning, management, implementation, and evaluation of health actions and services’ (CNPSI, 1986, p. 2).

It is important to underline the fact that the Indigenous interviewees highlighted Indigenous participation in all the debates and events related to the formulation of the Indigenous health subsystem:

*I can tell everyone that what exists today, in terms of laws, in terms of decrees, is an achievement of Indigenous people. But non-Indigenous society still ignores [this fact] because they think that the government gave us everything too easily. Many non-Indigenous people do not understand that it is a struggle and it is our right* (Zezinho Kaxarari).

This participation would be guaranteed in the new Indigenous Health Subsystem through the creation of a participatory structure in local, regional and national forums, respectively, the Local Council of Indigenous Health, the District Council of Indigenous Health (CONDISI) and the Intersectoral Commission on Indigenous Health (CISI) (Cornwall and Shankland, 2008).

As part of CIMI contributions to the 1st CNPSI, it was denounced that ‘the medical-preventive assistance applied by FUNAI is deficient in material, human, and qualitative aspects’ (CIMI, 1988, p. 11), and suggested that the recommendations of the 8th CNS be followed. UNI states that the new proposal should ‘be based on the technical-scientific principles of Primary Health Care’ (UNI, 1988, p. 9).

In the health reform movement, there was a process throughout the 1980s that sought to develop innovative health services (Escorel, 1999; Paim, 2008). During this period, the proposal emerged to organize health districts as a framework for the new health system. This proposal was influenced by the promotion of primary health care in the international context, and especially the strategy of Local Health Systems (*Sistemas Locales de Salud*: SILOS) by the Pan American Health Organization (PAHO) (Mendes, 1999). In Brazil, the SILOS proposal was redrafted as health districts (Mendes, 1999; Silva Junior, 2006).

In the definition of health districts, the emphasis is placed on the territorial dimension, which is both organizational and political, since it proposes sharing the decision-making process (Mendes, 1999; Silva Junior, 2006). Territory is understood as a ‘process territory, a space under permanent construction, a product of a social dynamic’ (Mendes, 1999, p. 166). Therefore, the discussion of health districts resonates with a key element of the struggle of Indigenous movements, namely the protection of Indigenous territories, considered as a fundamental right to health of Indigenous peoples (CIMI, 1988; UNI, 1988). In our view, this seems to be a good example of how policies are produced through the intersection of meanings (Bernstein, 2017).

Consequently, a health-care model based on territory had a strong affinity with earlier debates of the Indigenous movement. The health district’s proposal, which considers the specificities of each Indigenous territory, has been incorporated into diverse documents since 1989.

Additionally, in those early contributions to the new Indigenous health policy, UNI and CIMI emphasized the need for direct co-ordination with a Secretary linked to the Ministry of Health, and therefore in federal management. As a result, when the health reform movement replaced the central role of health districts with the idea of municipalization, and the 9th National Heath Conference (9th CNS) in 1992 was launched with the slogan ‘Municipalization is the way forward!’, there was a strong reaction from the Indigenous movement:

*The [9^th^ National Health Conference] was linked to the issue of the municipalization and decentralization of the national health system. And for the Indigenous movement, there was the certainty that municipalization was not the desired solution; they even made a banner declaring ‘Municipalization is not the way forward for Indigenous health*’ (Alba Figueroa).

The Indigenous movement historically identifies the municipal level as a major adversary in the struggle for territory, for this is the stratum where the social actors disputing their lands are located. Thus, for a long time it was established that Indigenous affairs should be a federal government responsibility (CIMI, 1988; UNI, 1988; Cunha, 2018). Immediately after the 9th CNS, there was a mobilization to organize the 2nd National Health Conference for Indigenous Peoples (2nd CNSPI), which was held in 1993. At this event, which involved major Indigenous participation, it was approved that ‘the model for Indigenous health care is based on the Indigenous Special Health District (DSEI)’ (CNSPI, 1993, p. 3), and that ‘responsibility for Indigenous health care is assigned to the Federal Government’ (CNSPI, 1993, p. 2). We identified the issue of municipalization as the main point of conflict between the Indigenous movement and the health reform, and this remains the case. However, the Indigenous movement was successful. On 23 September 1999, Law 9836 (the Arouca Law) created the ‘Indigenous Health Care Subsystem’ based on ‘Indigenous Special Health Districts’, and under the responsibility of the Ministry of Health.

## Conclusion

In this paper, we aimed to reconstruct the context, actors and discourses that Indigenous peoples and their allies mobilized in the pursuit of Indigenous rights to health in Brazil during the late decades of the 20th century. It is important to note that the politically progressive perspectives that characterized the debates on Indigenous health policy in Brazil emerged in the context of resistance to the dictatorship, embedded in principles such as social justice and democracy. It was a period of intense mobilization and collaboration between Indigenous leaders, anthropologists, health professionals and participants of the sanitary reform movement. Therefore, even though national health reform leaders were not necessarily directly involved in the struggles for Indigenous rights, the notion of a ‘holistic’ health perspective, social participation and reorganization of the health-care model made space to support the cause for these minorities.

Based on the interviews and documents, we have argued that the formulation of the Indigenous health policy was a long-term process, grounded in the context of struggles for the rights of Indigenous peoples in the 1980s, with the emergence of the Indigenous movement, and its intersections with the debates about sanitary reform in Brazil. Most studies on the trajectory of the Indigenous health policy in Brazil have not emphasized the role played by Indigenous and pro-Indigenous participation, particularly in the 1970s and 1980s. We found evidence that the fundamental document for the new Indigenous health policy, the 1st CNPSI report (CNPSI, 1986), was essentially based on the contributions of non-governmental organizations of both Indigenous (UNI, 1988) and non-Indigenous (CIMI, 1988) backgrounds. Particularly, the narratives of Indigenous leaders, as revealed in the interviews, point to their agency and reflective perspectives that effectively contributed to shape the new Indigenous health policy. Our interpretation is that the Brazilian case study we have explored in this paper is an example that health policy is far from ‘an entity defined from on high by decision makers’ (Shore and Wright, 2011, p. 24).

At this point in time, in 2020, nearly two decades after the establishment of the Indigenous Health Subsystem in Brazil, Indigenous peoples face daunting challenges on a multitude of fronts because of the current political situation in the country. As pointed out in a recent *Lancet* editorial, ‘Bolsonaro's presidency represents the most serious threat to Brazil's Indigenous population since the 1988 Constitution…’ (Anonymous, 2019). Recent government measures have greatly threatened the maintenance of the Indigenous Health Subsystem (Fraser, 2019). Once again, the Indigenous movement must call on its communities and organizations to fight for their right to health (APIB, 2019).
